# Social stigma of people with mental disorders and attitude to psychiatric treatment in Polish society

**DOI:** 10.1192/j.eurpsy.2023.993

**Published:** 2023-07-19

**Authors:** M. Burdzik

**Affiliations:** Department of Psychiatry, Center of Psychiatry in Katowice; Institute of Legal Sciences, University of Silesia, Katowice, Poland

## Abstract

**Introduction:**

People with mental disorders (MD) may experience social stigma in various spheres of their lives. This phenomenon is based on negative social beliefs and hostile perceptions about mental disorders. Stigma may lead to social exclusion and discrimination (unjust treatment compared to people without MD). It may also result in resistance to using professional medical help (psychiatrist, psychotherapist) by people who experience symptoms of MD.

**Objectives:**

The study aims to analyze the attitude of Polish society toward people with mental disorders and the attitude toward psychiatric treatment. The study investigates the correlation between the abovementioned attitudes and socioeconomic parameters of the respondents.

**Methods:**

The study was conducted on a group of 1,230 respondents with the anonymous, authorial questionnaire disseminated by the CAWI technique. The questionnaire consisted of 10 single-choice questions concerned with the socioeconomic parameters of the respondents (age, place of residence, education, gender) and their attitudes toward mental health problems. The chi-square test and Kendall’s tau-b correlation coefficient (τb) were used to analyze the correlations between the above parameters (with p<0,05).

**Results:**

Over 33% of respondents believes that people with MD are more aggressive than people who do not present this type of disorder. In turn, 16.4% of respondents admitted that they would feel uncomfortable in the presence of a person with mental disorders. There was no statistically significant correlation between the above beliefs and any socioeconomic parameter. Every tenth respondent would not hire a person with MD. Resistance to employment increased with the respondents’ age and level of education, whereas it decreased with the population of respondents’ domiciles. More than 17% of respondents would feel resistance to contacting with psychiatrist, and 4.1% of them already hide the fact of treatment from their family. The resistance to using psychiatric help was higher in villages and smaller towns than in bigger centers.

**Conclusions:**

The study shows negative attitudes towards people with MD are still relatively frequent in Polish society. The stereotypical perception of this group of entities is generally not dependent on any analyzed socioeconomic parameter. Such correlations exist only in some areas (employment). Because of the negative perception of MD, some people who struggle with these problems do not use a psychiatrist’s professional help. In effect, these entities remain undiagnosed and untreated. Treatment delay may lead to exacerbating symptoms, prolong treatment time (including the necessity of hospitalization), and increase its cost. Reducing the stigma is necessary for counteracting discrimination against people with mental disorders and improving the mental health condition of Polish society. It requires educational activities and appropriate legal regulations.

**Disclosure of Interest:**

None Declared

